# The eNOS-NO pathway attenuates kidney dysfunction via suppression of inflammasome activation in aldosterone-induced renal injury model mice

**DOI:** 10.1371/journal.pone.0203823

**Published:** 2018-10-03

**Authors:** Yuji Sogawa, Hajime Nagasu, Seiji Itano, Kengo Kidokoro, Shun’ichiro Taniguchi, Masafumi Takahashi, Hiroyuki Kadoya, Minoru Satoh, Tamaki Sasaki, Naoki Kashihara

**Affiliations:** 1 Department of Nephrology and Hypertension Kawasaki Medical School, Kurashiki, Okayama, Japan; 2 Department of Molecular Oncology, Shinshu University Graduate School of Medicine, Matsumoto, Nagano, Japan; 3 Division of Inflammation Research, Center for Molecular Medicine, Jichi Medical University, Shimotsuke, Tochigi, Japan; UCL Institute of Child Health, UNITED KINGDOM

## Abstract

Hypertension causes vascular complications, such as stroke, cardiovascular disease, and chronic kidney disease (CKD). The relationship between endothelial dysfunction and progression of kidney disease is well known. However, the relationship between the eNOS–NO pathway and chronic inflammation, which is a common pathway for the progression of kidney disease, remains unexplored. We performed in vivo experiments to determine the role of the eNOS–NO pathway by using eNOS-deficient mice in a hypertensive kidney disease model. All mice were unilateral nephrectomized (Nx). One week after Nx, the mice were randomly divided into two groups: the aldosterone infusion groups and the vehicle groups. All mice also received a 1% NaCl solution instead of drinking water. The aldosterone infusion groups were treated with hydralazine to correct blood pressure differences. After four weeks of drug administration, all mice were euthanized, and blood and kidney tissue samples were collected. In the results, NLRP3 inflammasome activation was elevated in the kidneys of the eNOS-deficient mice, and tubulointerstitial fibrosis was accelerated. Suppression of inflammasome activation by knocking out ASC prevented tubulointerstitial injury in the eNOS knockout mice, indicating that the eNOS–NO pathway is involved in the development of kidney dysfunction through acceleration of NLRP3 inflammasome in macrophages. We revealed that endothelial function, particularly the eNOS–NO pathway, attenuates the progression of renal tubulointerstitial injury via suppression of inflammasome activation. Clinically, patients who develop vascular endothelial dysfunction have lifestyle diseases, such as hypertension or diabetes, and are known to be at risk for CKD. Our study suggests that the eNOS–NO pathway could be a therapeutic target for the treatment of chronic kidney disease associated with endothelial dysfunction.

## Introduction

Hypertension causes vascular complications, such as stroke, cardiovascular disease[1, 2], and chronic kidney disease (CKD)[3, 4]. Endothelial dysfunction is an initial step for the development of these complications, including CKD. In patients with CKD, high blood pressure and the renin-angiotensin-aldosterone system induce endothelial dysfunction[5] through impairment of the endothelial nitric oxide synthase–nitric oxide (eNOS–NO) pathway. The uncoupling of eNOS and the activation of NADPH oxidase are major sources of reactive oxygen species (ROS)[6], and we have previously reported the relationship between ROS and endothelial dysfunction in the progression of hypertension-induced kidney disease[7, 8]. Several recent studies on cardiovascular diseases have reported that endothelial dysfunction has a pivotal role in the progression of these diseases[9].

Chronic inflammation is a well-known pathway to the development of kidney disease; however, the relationship between endothelial dysfunction and chronic inflammation in CKD remains unclear. The eNOS–NO pathway, in particular, plays a pivotal role in the expansion of inflammation because the NO generated by eNOS induces the vascular permeability needed for the infiltration of macrophages associated with infectious diseases. In general, macrophages play a central role in innate immune protection through the clearance of infective pathogens and through the repair of tissue injury that occurs, in part, as a consequence of this response[10]. The infiltration of macrophages causes the renal inflammation that accompanies most forms of CKD, including hypertensive kidney disease[11]. In CKD, macrophages are crucial immune cells responsible for the development of chronic inflammation and tubular cell injury via ROS production[12, 13]. Studies on hypertensive animal models have confirmed that ROS can further exacerbate kidney injury by proinflammatory reactions involving the cytokines IL-1β and IL-18[14, 15]. These inflammatory cytokines are generated following inflammasome activation in macrophages, and this has prompted our focus on the role of inflammasome activation in hypertensive kidney disease.

Inflammasomes are generated as an innate immune response to either exogenous pathogens or endogenous danger signals. They are multi-protein complexes that include a nucleotide-binding domain and NOD-like receptors (NLRs) that contain leucine-rich repeats. The NOD-like receptor family member, NLRP3, and an adaptor apoptosis-associated speck-like protein with a caspase recruitment domain (ASC) within inflammasomes activate caspase 1, which, in turn, promotes the maturation of proinflammatory cytokines, including IL-1β and IL-18. Inflammasomes, especially NLRP3 inflammasomes, have been implicated in inflammation in a mouse kidney disease model[16, 17].

We have previously reported that inflammasome activation is important in the infiltration of proinflammatory M1 macrophages into interstitial lesions[18]. We have focused on NO following the discovery by Kairui Mao et al.[19] of the role of NO generated from iNOS in the regulation of the NLRP3 inflammasome and a subsequent report suggesting the regulation of tuberculosis immunopathology by NO through the inhibition of NLRP3 inflammasome-dependent IL-1β processing[20]. Both reports revealed that NO inhibits ASC oligomerization in nigericin-stimulated macrophages, suggesting that the NO generated from iNOS might act post-translationally to regulate inflammasome function. However, the relationship between the eNOS–NO pathway and the inflammasome, especially NLRP3 inflammasomes, remains unexplored.

In this study, we used eNOS-deficient mice and generated eNOS-ASC double knockout mice to examine whether the eNOS–NO pathway can attenuate inflammasome activation in the progression of kidney disease.

## Materials and methods

### Animals

The experimental protocols (nos. 16–108, 16-33-1 and 17–107) were approved by the Animal Research Committee of Kawasaki Medical School. These were based on the National Institutes of Health Guide for the Care and Use of Laboratory Animals (NIH Publication No. 80–23, revised 1996). Eight-week-old male C57B/6 J mice weighing 20 to 30 g at the beginning of the study were designated as wild type (WT). WT and *eNOS* knockout mice (eNOS KO) were purchased from Jackson Laboratory (Bar Harbor, ME) [21]. *Asc* homozygous knockout (ASC KO) mice were kindly provided by M. Takahashi (Jichi Medical University, Shimotsuke, Japan)[22]. Then, eNOS/ASC DKO were generated by intercrossing eNOS KO and ASC KO mice.

The mice were housed in a temperature- and humidity-controlled room with a 14:10 h light–dark cycle and were fed standard laboratory animal chow with free access to tap water. All mice were unilaterally nephrectomized (Nx). One week after Nx, the mice were randomly divided into two groups (*n = 8 per group*): the aldosterone infusion groups and the vehicle groups. All mice also received a 1% NaCl solution instead of drinking water. Aldosterone (0.25 mg/kgBW/day, Sigma- Aldrich, Merck KGaA) dissolved in distilled water was used to fill an Alzet osmotic minipump (Durect, Cupertino, CA, USA). The pump was implanted subcutaneously to infuse aldosterone[11]. In the experiment comparing the WT and eNOSKO group, the aldosterone infusion groups were treated with hydralazine (30 mg/kg BW/day) (Sigma-Aldrich, Tokyo, Japan) (23) to correct blood pressure differences. In the experiment comparing the eNOSKO and eNOS/ASC DKO groups, hydralazine was not administered because blood pressure was not different in these mice. After four weeks of drug administration, all mice were euthanized, and blood and kidney tissue samples were collected.

### Physiological and biochemical measurement

Physiological parameters were measured just before the mice were sacrificed at 12 weeks old.

Their body weight was determined, and blood pressure was measured using the tail-cuff method (BP-98A; Softron, Tokyo, Japan). Blood samples were obtained using a 21-gauge needle inserted into the right atrium of mice that fasted for 24 h.

### Histology, immunohistochemistry, and immunofluorescence

Right kidney tissue was fixed in 4% paraformaldehyde and embedded in paraffin for histological analysis. Tissue sections (approximately 2 μm thick) were deparaffinized and stained by Masson trichrome staining. Immunochemical staining was conducted on the deparaffinized kidney sections (4 μm thick) by heating in a microwave at 500 W for 15 min for antigen retrieval, followed by incubating overnight with an antibody against collagen IV (Abcam, Cambridge, MA, USA), KIM-1 (KCA031610A, R&D Systems, Minneapolis, MN, USA). The primary antibody was detected using the Histofine Simple Stain MAX‑PO kit (Nichirei Corporation, Tokyo, Japan) and 3,3'‑diaminobenzidine (Sigma‑Aldrich; Merck KGaA).

The severity of tubulointerstitial injury was evaluated by examining 10 optical fields in randomly selected tissue samples. The images of scarred areas (stained blue with Masson’s trichrome) and areas with positive staining for KIM-1, COLAIV (brown) were quantified using a color image analyzer (KYEENCE). The glomeruli, tubules, and blood vessels of the cortex were excluded. The results were presented as percentages of the relative volume of the scanned interstitium[23].

Glomerular change was evaluated by Periodic acid–Schiff (PAS) staining. Glomerular sclerosis score (0–4) to assess the degree of glomerular damage: grade 0, normal glomeruli; grade 1, sclerotic area up to 25%; grade 2, sclerotic area 25 to 50% (moderate sclerosis); grade 3, sclerotic area 50 to 75%; grade 4, sclerotic area 75 to 100% (severe sclerosis). The glomerulosclerotic index was calculated using the following formula: GSI = (1 × A1 + 2 × A2 + 3 × A3 + 4 × A4)/(A0 + A1 + A2 + A3 + A4), where Ax is the number of glomeruli in each grade of glomerulosclerosis.

### Quantitative real-time reverse transcriptase-PCR

Total mRNA extraction and real-time reverse transcription quantitative PCR (RT-qPCR) were performed, as described previously[18]. The primers and probes for the TaqMan analysis were designed using sequence information from GenBank (National Institutes of Health, Bethesda, MD, USA) and Primer3 online software (http://frodo.wi.mit.edu/primer3/).

The primer and probe sequences are listed in S1 Table. TaKaRa Premix Ex Taq (Takara Bio, Inc., Otsu, Japan), with a final reaction volume of 20 μl, was used for the TaqMan probe-based quantitative reverse transcription PCR reaction, which was performed on an Applied Biosystems 7500 Fast Real-Time PCR System (Applied Biosystems; Thermo Fisher Scientific, Inc.). The level of mRNA expression in each sample was quantified using the absolute quantification standard curve method. The plasmid cDNA of each gene was used to prepare the absolute standards. The concentration was measured using the A260/A280 ratio, which was converted to the number of copies by using the molecular weight of the DNA. Each mRNA expression level was normalized to that of the housekeeping 18s ribosomal RNA gene.

### Western blot analysis

Kidney and cell lysates were prepared, as described previously. The protein samples (50 μg per lane) were subjected to immunoblotting analysis with antibodies against IL-1β (Abcam) and glyceraldehyde phosphate dehydrogenase (GAPDH; Santa Cruz Biotechnology). Signals were detected using an enhanced chemiluminescence system (GE Healthcare Japan, Tokyo, Japan)[24].

### Cell culture

Bone marrow-derived macrophages were used for the in vitro assays. Bone marrow was isolated from femur bone and bone marrow cells. Cell cultures were prepared using an L929-conditioned medium, including media containing the macrophage colony-stimulating factor [25]. To activate the NLRP3 inflammasome, bone marrow-derived macrophages (BMDMs) were primed for 3 h with ultrapure LPS (InvivoGen, San Diego, CA, USA), followed by stimulation with ATP (5 mM) 30 min before the cell lysates and supernatants were harvested. GSNO (500μM, Sigma-Aldrich) and BAY 41–2272; Bay(10μM, Sigma-Aldrich) were treated at the same time as LPS. Aldosterone (1mM, Sigma-Aldrich) was stimulated for 18h after priming for 3h with LPS.

IL-1β in supernatants was detected using ELISA (R&D Systems, Minneapolis, MN, USA).

### Statistical analysis

Data are expressed as the mean ± standard error of the mean (SEM). All values are expressed as the SEM. Statistical analyses were calculated using GraphPad Prism7 software (GraphPad Software, La Jolla, CA, USA). Statistical significance was evaluated using one-way ANOVA with Tukey–Kramer post hoc tests for comparisons among multiple groups. P values less than 0.05 were considered statistically significant.

## Results

### eNOS deficiency exacerbates tubulointerstitial injury in a hypertensive mouse model

The physiological characteristics of the four groups are listed in Table 1. No significant difference was noted in the body weight and serum creatinine of the four groups. The systolic blood pressure was significantly higher in the eNOS KO-vehicle groups than in the other groups. However, no change in blood pressure was observed in the aldosterone groups after treatment with hydralazine. We confirmed that Hydralazine did not suppress inflammasome activation in primary bone marrow-derived macrophages (BMDMs) (S1 Fig). Blood pressure was significantly higher in eNOSKO-Ald group at baseline compared with WT-Ald. Thereafter, blood pressure in eNOSKO-Ald group was adjusted to the same level as in WT-Ald group (S2 Fig). Urinary albumin excretion was markedly increased in the eNOS KO-Ald groups compared with the WT-Ald groups. Glomerular change was assessed by PAS staining and sclerosis scores (0–4) (S3 Fig). Glomerular injury was exacerbated in eNOS-Ald group as compared with that in the WT-Ald group.

**Table 1:** Physiological characteristics at 4weeks after aldosterone infusion.

	WT-vehicle			WT-Ald			eNOSKO-vehicle			eNOSKO-Ald		
BW(g)	25.3	±	0.5	26.4	±	0.5	25.4	±	0.6	25.1	±	0.3
s-Cr (mg/dL)	0.18	±	0.01	0.15	±	0.01	0.19	±	0.02	0.15	±	0.01
SBP (mmHg)	105	±	2	97	±	3	128	±	5^1^	92	±	3
DBP (mmHg)	69	±	2	59	±	3	78	±	5	53	±	2
UAE (ng/g/CRN)	30.8	±	11.1	24.6	±	5.0	33.9	±	7.3	57.6	±	9.8^2^

Abbreviations: BW, body weight; S-Cre, serum creatinine; SBP, systolic blood pressure; DBP, diastolic blood pressure; u-CRN, urinary creatinine; UAE, urinary albumin excretion. Data are expressed as mean

± SEM.

^1^P < 0.05 vs. WT-vehicle

^2^P < 0.05 vs. WT-Ald

**Fig 1:**
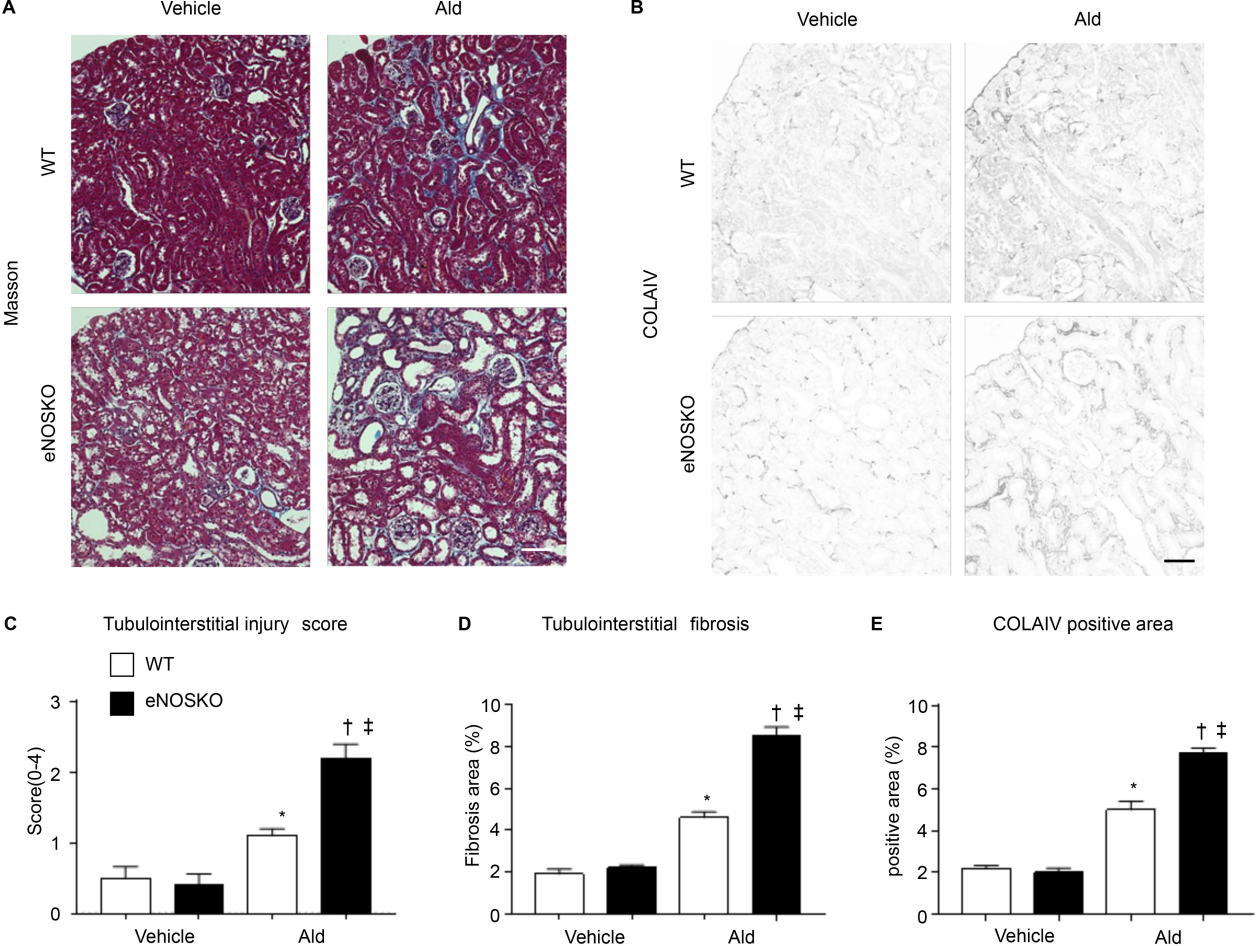
Histological fibrotic changes in the tubulointerstitium. (**A**) Masson-trichrome (Masson) staining showing renal tubulointerstitial morphology. Bar = 100 μm. (**B**) Collagen IV (COLAIV) staining showing tubule interstitial fibrotic change. Bar = 100 μm. WT-vehicle, control WT mice (n = 8); WT-Ald, WT-aldosterone infusion mice (n = 8); eNOSKO-vehicle, control eNOS KO mice (n = 8); eNOSKO-Ald, eNOS KO-aldosterone infusion mice (n = 8). (**C**) Tubulointerstitial injury score (0–4), (**D**) tubulointerstitial fibrosis area (%), and (**E**) COLA IV positive area (%). Data are shown as mean ± SEM. *P < 0.05 vs. WT-vehicle. ^†^P < 0.05 vs. WT-Ald. ^‡^P <0.05 vs. eNOSKO-vehicle.

We assessed tubulointerstitial injury by histological evaluation of kidney tissue samples (Fig 1). The extent of fibrosis was evaluated with Masson’s trichrome staining and collagen IV staining (Fig 1A and 1B). The tubular damage score was significantly higher in the eNOS KO-Ald groups than in the WT-Ald groups (Fig 1C). The Masson-positive area significantly increased in the WT-Ald groups compared with the WT-vehicle groups (Fig 1D). Immunohistochemical evaluation indicated a similar increase in the collagen IV positive area in the WT-Ald groups (Fig 1E). These changes were more severe in the eNOS KO-Ald groups, and the Masson staining was more extensive. Nitrotyrosine staining, which is a marker of ROS, was performed to determine the oxidative status. The percentage of nitrotyrosine-positive areas increased in the eNOSKO-Ald groups as compared with that in the WT-Ald groups (S4 Fig).

**Fig 2:**
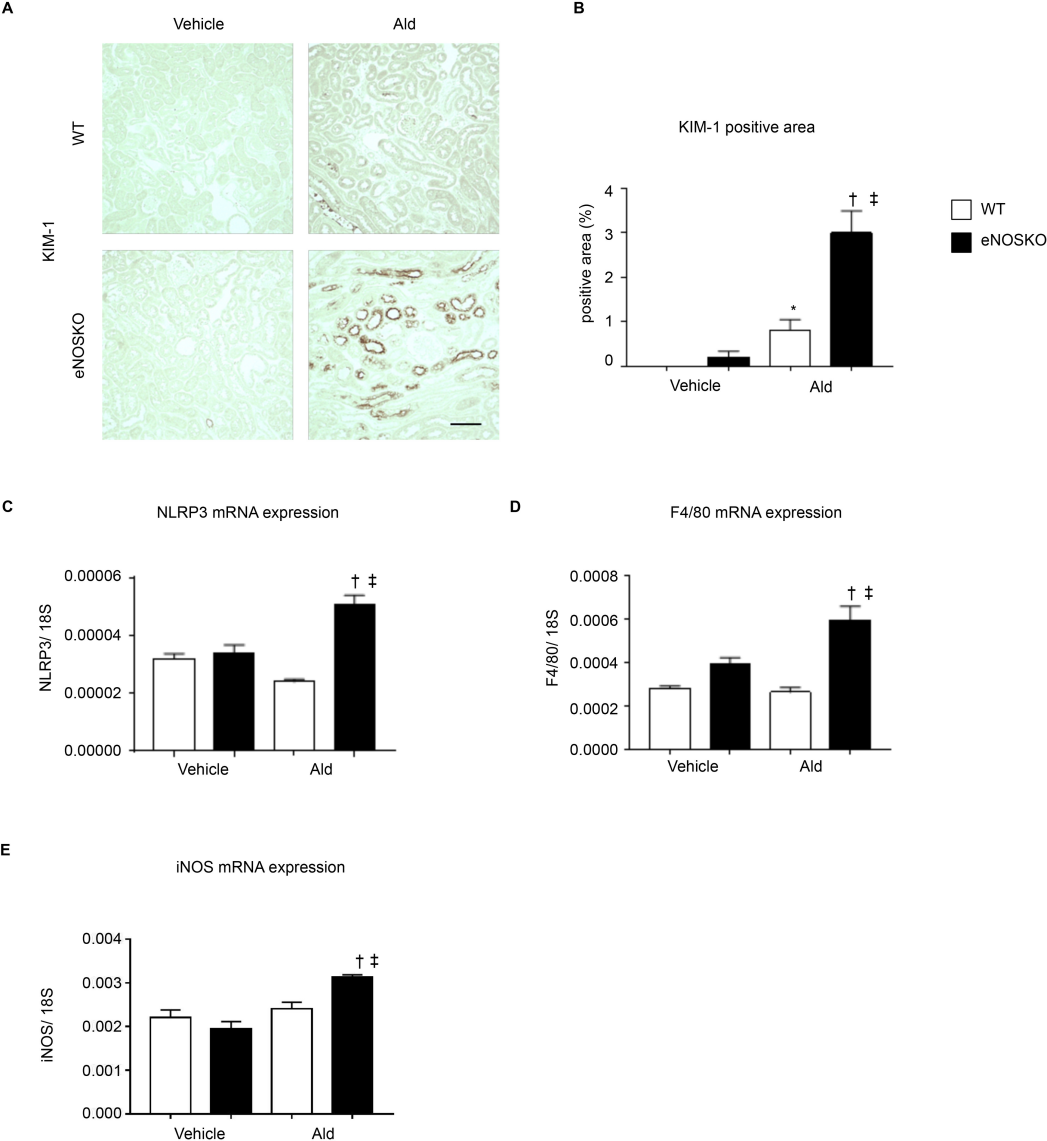
Inflammatory changes in the tubulointerstitium. (**A**) Kidney injury molecule-1 (KIM-1) staining showing renal inflammatory injured cells. Bar = 100 μm. (**B**) KIM-1 positive area (%). (**C**) mRNA expression of F4/80, macrophage-specific antigen. (**D**) mRNA expression of NLRP3, nucleotide-binding oligomerization domain, leucine-rich repeat, and pyrin domain-containing protein (NLRP) 3. (**E**) mRNA expression of iNOS, a marker of proinflammatory macrophages. Data are shown as mean ± SEM. *P < 0.05 vs. WT-vehicle. ^†^P < 0.05 vs. WT-Ald. ^‡^P <0.05 vs. eNOSKO-vehicle.

We also evaluated tubular injury by staining for kidney injury molecule 1 (KIM-1) and measuring the KIM-1 positive area (Fig 2A). The KIM-1 positive area was larger in the WT-Ald groups than in the WT-vehicle groups (Fig 2B), and the eNOS KO groups showed an even greater marked increase (Fig 2B). The mRNA levels of inflammation-related genes, such as F4/80, NLRP3 and iNOS were significantly higher in the eNOS KO-Ald groups than in the WT-Ald groups (Fig 2C and 2E). A deficiency in the eNOS-NO pathway resulting in endothelial dysfunction exacerbated tubulointerstitial inflammation or fibrosis after aldosterone infusion, and this effect was independent of systemic blood pressure.

### Inflammasome activation in macrophages affects tubulointerstitial injury in the hypertensive mouse model

**Fig 3:**
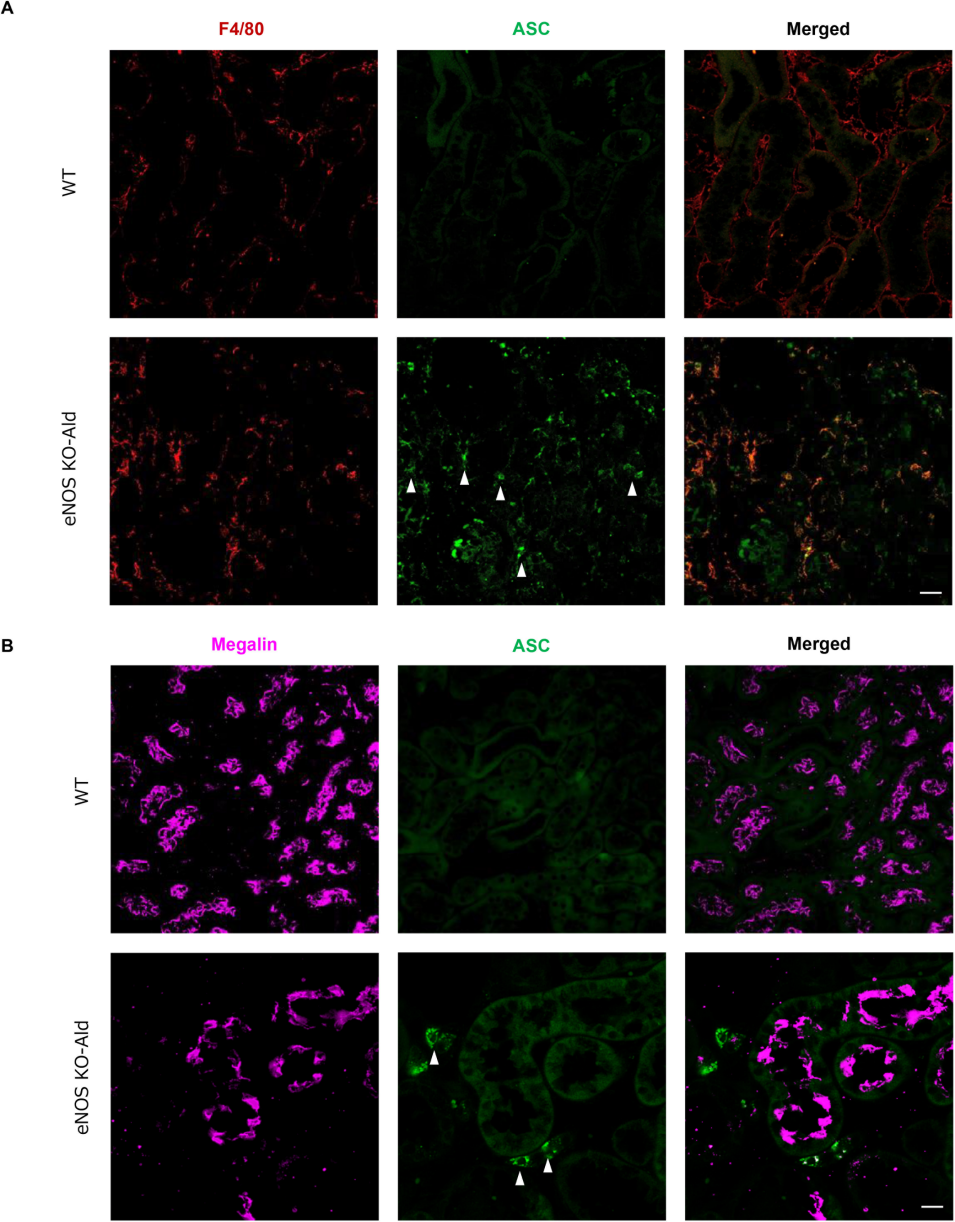
Inflammasome activation in infiltrated macrophages, not in tubular cells. (**A**) Double-immunofluorescence analysis of F4/80, ASC, and merged images in WT and eNOSKO-Ald, eNOS KO-aldosterone infusion mice. ASC, apoptotic speck protein containing a caspase recruitment domain, as a marker of inflammasome activation. F4/80, marker of macrophage. Bar = 20 μm. (**B**) Double-immunofluorescence analysis of F4/80, ASC, and merged images in WT and eNOSKO-Ald, infusion mice. Megalin, marker of tubular cells. Bar = 20 μm.

We identified the site of inflammasome activation after aldosterone infusion by immunofluorescence staining of the kidney tissues of the eNOS KO-Ald groups (Fig 3). F4/80 was used as a marker of macrophages. Megalin was chosen as a marker of proximal tubular cells, and ASC was selected as a key component in inflammasome activation. ASC staining was mainly localized in the macrophages (Fig 3A), but megalin was not co-stained with ASC (Fig 3B), suggesting that inflammasome activation in macrophages affected the progression of tubulointerstitial injury in the aldosterone infusion model.

### Tubulointerstitial damage and chronic inflammation were suppressed in eNOS-ASC double knockout mice

**Fig 4:**
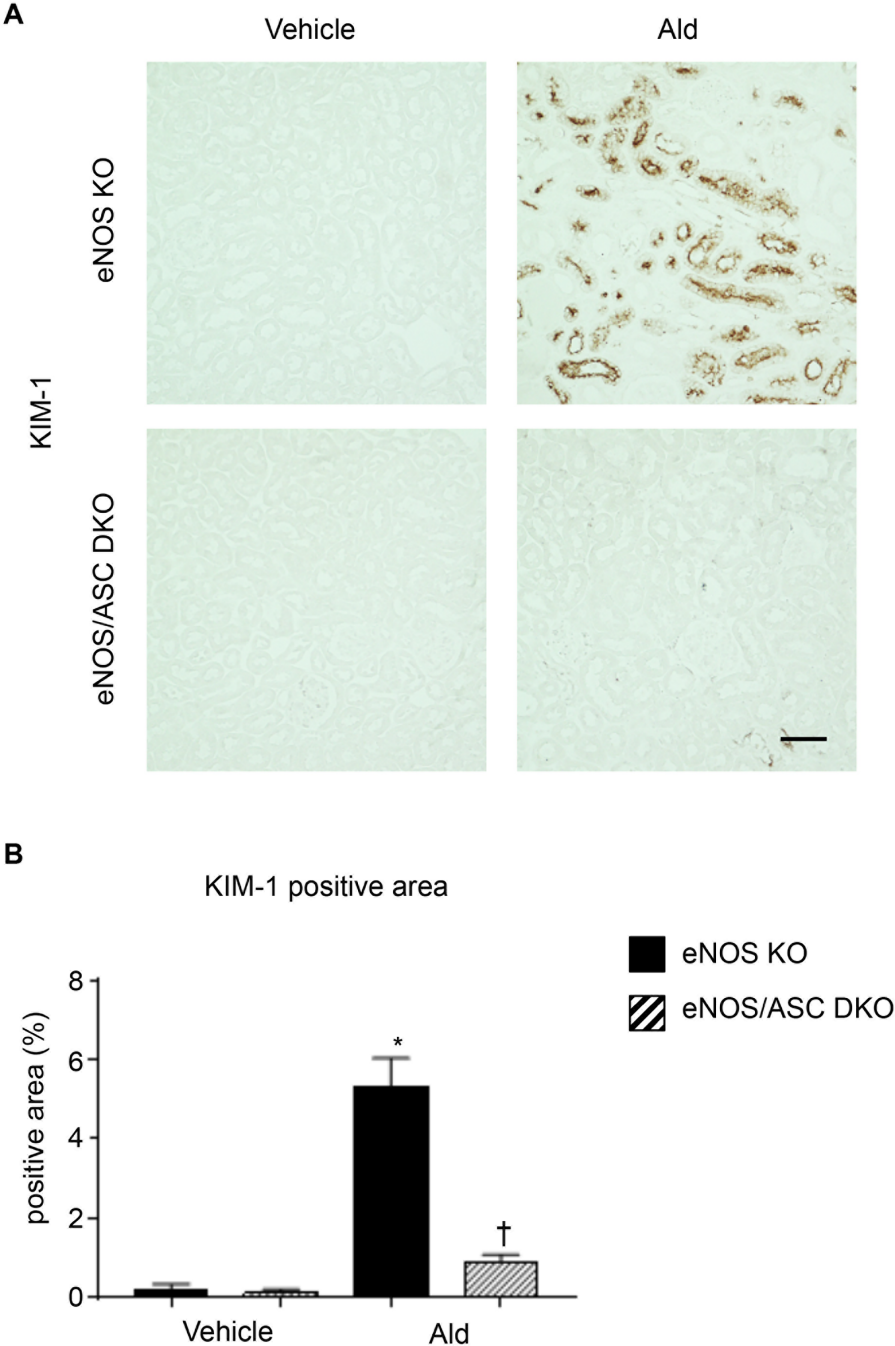
Tubular cell injury by aldosterone prevented in eNOS/ASC DKO mice. (**A**) KIM-1 staining showing renal inflammatory injured cells. Bar = 100 μm. (**B**) KIM-1 positive area (%). Data are shown as mean ± SEM. *P < 0.05 vs. eNOS KO-vehicle. ^†^P < 0.05 vs. eNOS KO-Ald.

We investigated whether the progression of tubulointerstitial injury in eNOS KO-Ald mice depended on inflammasome activation. We evaluated inflammasome activation by generating eNOS/ASC double knockout mice (eNOS/ASC DKO) and administering aldosterone. The physiological characteristics of the four groups are listed in Table 2 and then evaluating tubular injury by KIM-1 immunostaining. The eNOS KO-Ald groups showed a significantly larger KIM-1 positive area compared with the eNOS-vehicle groups, whereas the eNOS/ASC DKO-Ald groups showed a significantly decreased KIM-1 positive area compared with the eNOS KO-Ald groups (Fig 4).

**Table 2:** Physiological characteristics at 4weeks after aldosterone infusion.

	eNOSKO						eNOS/ASC DKO					
	vehicle			Ald			vehicle			Ald		
BW (g)	25.1	±	0.4	23.7	±	0.6	25.7	±	1.8	25.5	±	1.2
s-Cr (mg/dL)	0.15	±	0.01	0.19	±	0.02	0.15	±	0.01	0.15	±	0.01
SBP (mmHg)	132	±	6	150	±	3	128	±	9	143	±	4
DBP (mmHg)	84	±	12	94	±	7	79	±	10	84	±	6

Abbreviations: BW, body weight; S-Cre, serum creatinine; SBP, systolic blood pressure; DBP, diastolic blood pressure. Data are expressed as mean ± SEM.

**Fig 5:**
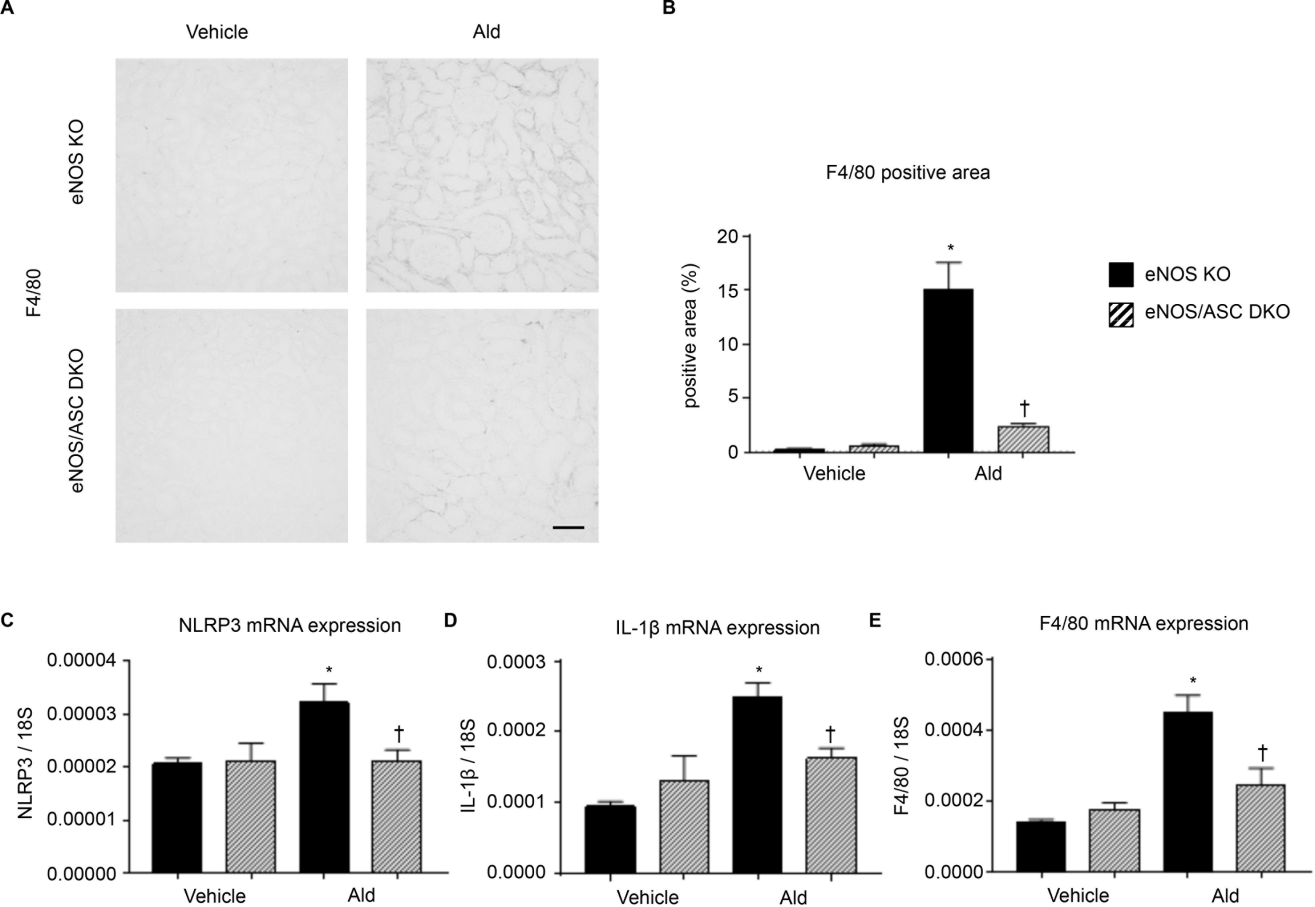
Inflammatory changes in eNOS/ASC DKO mice. (**A**) F4/80 staining showing infiltrated macrophages. Bar = 100 μm. (**B**) F4/80 positive area (%). eNOS KO-vehicle, control eNOS KO mice (n = 8); eNOS KO-Ald, eNOS-aldosterone infusion mice (n = 8); eNOS/ASC DKO-vehicle, control eNOS/ASC DKO mice (n = 8); eNOS/ASC DKO-Ald, eNOS/ASC DKO-aldosterone infusion mice (n = 8). (**C**) mRNA expression of NLRP3. (**D**) mRNA expression of IL-1β. (**E**) mRNA expression of F4/80. Data are shown as mean ± SEM. *P < 0.05 vs. eNOS KO-vehicle. ^†^P < 0.05 vs. eNOS KO-Ald.

Macrophage infiltration was assessed by F4/80 immunostaining (Fig 5A). The eNOS KO-Ald groups showed a significantly increased F4/80 positive area compared with the vehicle groups (Fig 5B). This change because of aldosterone infusion was significantly lower in the eNOS/ASC DKO groups (Fig 5A and 5B). The eNOS KO groups showed a significantly increased expression of the inflammasome-related genes *caspase 1*, *IL-1 β*, and *IL-18* after aldosterone infusion, whereas these changes were significantly suppressed in the eNOS/ASC DKO groups (Fig 5C and 5E).

**Fig 6:**
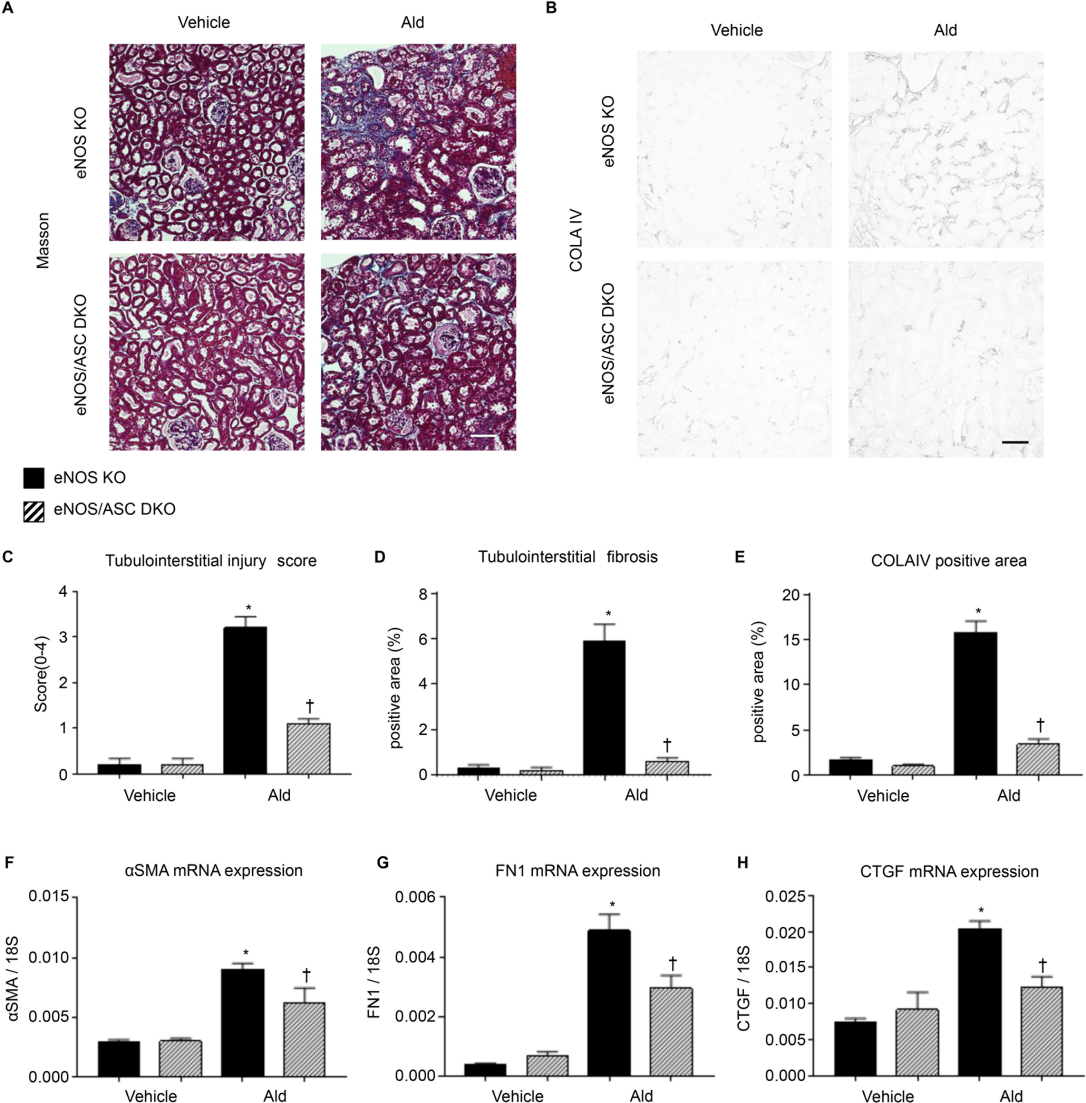
Fibrotic changes in eNOS/ASC DKO mice. (**A**) Masson staining showing renal tubulointerstitial morphology. Bar = 100 μm. (**B**) COLA IV staining showing tubule interstitial fibrotic change. Bar = 100 μm (**C**) Tubulointerstitial injury score (0–4), (**D**) tubulointerstitial fibrosis area (%), and (**E**) COLA IV positive area (%). (**F**) mRNA expression of alpha-smooth muscle actin, αSMA. (**G**) mRNA expression of fibronectin 1, FN1. (**H**) mRNA expression of connective tissue growth factor, CTGF. Data are shown as mean ± SEM. * P < 0.05 vs. eNOS KO-vehicle. ^†^P < 0.05 vs. eNOS KO-Ald.

Tubulointerstitial fibrosis was assessed by Masson staining and collagen IV staining (Fig 6A and 6B). Tubular injury was significantly exacerbated in the eNOS KO-Ald groups (Fig 6C) but was significantly reduced in the eNOS/ASC DKO groups (Fig 6C). Fibrosis was significantly suppressed in the eNOS/ASC DKO-Ald groups compared with the eNOS KO-Ald groups (Fig 6D and 6E).

The expression of the fibrosis-related genes alpha-smooth muscle actin (αSMA), fibronectin 1 (FN1), and connective tissue growth factor (CTGF) was significantly increased in the eNOS KO-Ald groups compared with the eNOS KO-vehicle groups, in agreement with the histological findings. Expression of these genes was significantly suppressed in the eNOS/ASC DKO groups (Fig 6F and 6H).

### NO, but not the sGC stimulator, suppresses inflammasome activation in macrophages

**Fig 7:**
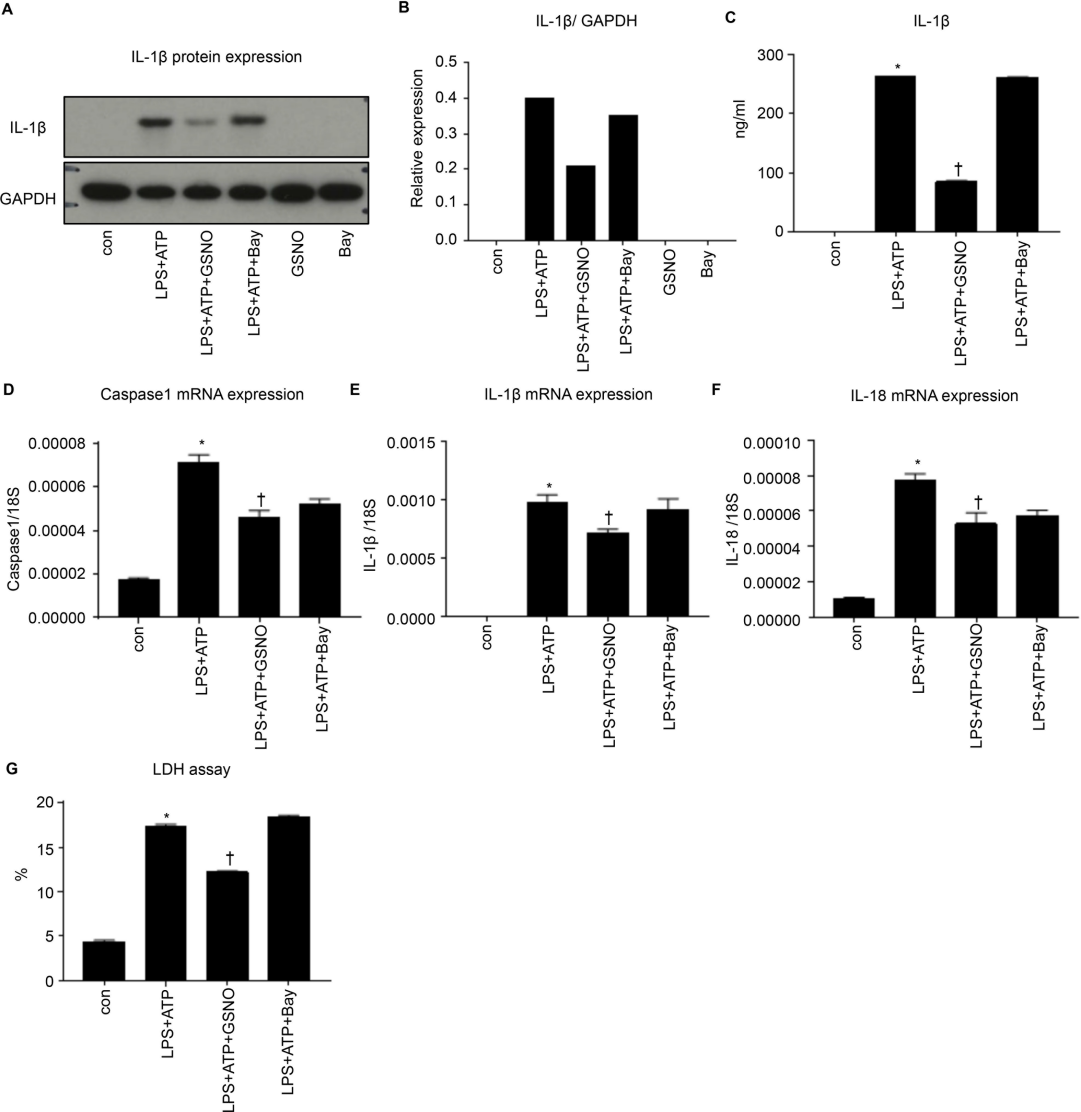
NO directly regulates inflammasome activation in primary cultured macrophages. In an in vitro experiment, inflammasome activation was evaluated in primary bone marrow-derived macrophages (BMDMs). Cont, BMDMs without stimulation; LPS+ATP, stimulated with ATP after LPS priming; LPS+ATP+GSNO, treated with NO donor, GSNO; LPS+ATP+Bay, treated with sGC stimulator, Bay. (**A**) Western blot analysis for IL-1β in macrophages. (**B**) Relative expression of IL-1β protein expression to GAPDH protein expression. (**C**) The levels of interleukin-1β (IL-1β) were detected with enzyme-linked immunosorbent assay (ELISA) in the supernatant. (**D**) mRNA expression of caspase 1, (**E**) mRNA expression of IL-1β, (**F**) mRNA expression of IL-18. (**G**) Lactate dehydrogenase (LDH) assay of the supernatant. Data are shown as mean ± SEM. *P < 0.05 vs. control. ^†^P < 0.05 vs. LPS+ATP.

We examined the effects of the absence of eNOS in inflammasome activation in macrophages by using primary in vitro cultures of BMDMs. After incubation with LPS, the BMDMs were administered ATP to activate NLRP3 inflammasome (Fig 7). We used GSNO as an NO donor and Bay as an sGC stimulator.

The protein level of IL-1β was elevated by LPS+ATP stimulation, but it was suppressed by LPS+ATP+GSNO stimulation (Fig 7A and 7B). Conversely, it was not reduced by LPS+ATP+Bay stimulation. When the BMDMs were stimulated with GSNO and Bay alone, the IL-1β levels did not increase. The IL-1β concentration in the supernatant was significantly increased after stimulation with LPS+ATP compared with the non-stimulated cells (Fig 7C), but GSNO treatment suppressed LPS+ATP–stimulated IL-1β secretion into the supernatant (Fig 7C). However, the cells treated with LPS+ATP+Bay showed no suppression of IL-1β stimulation by LPS+ATP (Fig 7C).

The expression of the inflammasome-related genes *caspase 1*, *IL-1 β*, and *IL-18* was significantly elevated following LPS+ATP stimulation, and these elevations were suppressed in the cells treated with GSNO. By contrast, the Bay treatment did not prevent the increased expression of these genes (Fig 7D and 7F).

Cell death after inflammasome activation was evaluated with the LDH assay. In the LPS+ATP group, LDH activity was significantly increased compared with that in the untreated group. The LPS+ATP+GSNO treated group showed a significant suppression of cell death compared with the LPS+ATP group. The LPS+ATP+Bay treatment did not suppress cell death compared with the LPS+ATP treatment (Fig 7G).

## Discussion

The purpose of this study was to determine whether endothelial dysfunction accelerates inflammasome activation and exacerbates tubulointerstitial injury. We found that kidney injury was exacerbated via inflammasome activation in eNOS-deficient mice treated with aldosterone. NLRP3 inflammasome activation was elevated in the kidneys of eNOS-deficient mice, and it accelerated tubulointerstitial fibrosis. Suppression of inflammasome activation by knocking out ASC prevented tubulointerstitial injury in eNOS KO mice, indicating that the eNOS–NO pathway is involved in the development of kidney dysfunction through acceleration of the NLRP3 inflammasome in macrophages. Our in vitro data suggested that NO directly suppresses inflammasome activation in BMDMs.

In general, the eNOS–NO pathway accelerates local inflammation by increasing vascular permeability to allow access of inflammatory cells, such as macrophages, to the infected tissues. One of the mechanisms by which NO contributes to increased permeability is through the cooperation between NO and VEGF signaling at adherens junctions, as several studies have demonstrated that eNOS is essential for VEGF-mediated changes in microvascular permeability[26, 27]. The NO generated by eNOS regulates many biological processes through cGMP-dependent mechanisms[28], and β-catenin is a substrate for S-nitrosylation by NO. Stimulation of endothelial cells with VEGF induces S-nitrosylation of β-catenin, and this reaction is dependent on the expression and activity of eNOS, with the end result being an increase in vascular permeability[29]. Therefore, eNOS-derived NO controls vascular permeability in vivo through multiple actions, including modulation of blood flow in post capillary venules, protein kinase G activation, and NO-mediated chemical modifications of junctional proteins.

By contrast, endothelial cell dysfunction is an initial step in the vascular complications occurring concomitantly with inflammation in hypertension. Endothelial cells have several roles in the kidney, but the eNOS–NO pathway is the most important pathway in endothelial function. Many reports have revealed that the impaired availability of NO, caused by either inhibition or genetic defects of eNOS, promotes the progression of renal dysfunction[7, 30]. Our data also suggest that eNOS deficiency accelerated the infiltration of macrophages, as chronic inflammation in interstitial lesions was exacerbated in the aldosterone-induced hypertensive model mice. These data suggested that a lack of generation of NO by endothelial cells exacerbates interstitial inflammation and fibrosis.

We examined the underlying mechanism by focusing on inflammasome activation in the kidney because excessive aldosterone directly activates the inflammasome in infiltrating macrophages and promotes tubulointerstitial fibrosis[11]. In general, ROS are considered important in inflammasome activation. However, in the present study, there was no significant difference between the WT-Ald and eNOS KO-Ald groups. This result suggested that a deficiency in the eNOS-NO pathway did not affect ROS in this model. However, as we investigated the presence of only one ROS marker, we are unable to conclude whether ROS plays an important role in inflammasome activation in hypertensive kidney disease. Next, the eNOS-ASC double knockout mice were generated in the present study to determine whether the loss of NO derived from eNOS exacerbates tubulointerstitial injury through inflammasome activation. We demonstrated an attenuation of tubular injury and inflammation observed in the eNOS KO mice by also knocking out ASC, and we saw no tubulointerstitial inflammation in the eNOS/ASC DKO mice following aldosterone infusion. However, this inflammasome activation was prevented by deletion of ASC, thereby confirming the relationship between the eNOS–NO pathway and inflammasome activation in vivo.

We were unable to determine which cells were responsible for inflammasome activation, but most reports have suggested that macrophages are the main source of inflammasome activation. We have also reported the role of inflammasome activation in maintaining the infiltration of M1 macrophages (which are pro-inflammatory macrophages) in unilateral ureter obstruction[18]. Other reports have suggested that extracellular ATP, released from renal tissue after ureteral obstruction, activates the NLRP3 inflammasome by binding to the P2X7 receptor to induce potassium efflux and ROS production in the collecting duct epithelial cells[31].

How the inflammasome is regulated by NO is an important unresolved question. The NO generated by iNOS is a signaling molecule that plays a key role in the pathogenesis of inflammation. NO is pro-inflammatory at low concentrations and induces vasodilatation and the recruitment of neutrophils. By contrast, at high concentrations, it down-regulates adhesion molecules, suppresses activation, and induces apoptosis of inflammatory cells[32]. However, the mechanism by which NO regulates the inflammasome in macrophages is unclear.

We addressed this question by using in vitro assays and BMDMs, and we found evidence to suggest that NO can directly inhibit inflammasome activation in macrophages. NO is involved in several physiological processes, including regulation of cellular proliferation, hypertrophy, and survival. NO is also known to activate cGMP-dependent protein kinase G (PKG) by activating downstream soluble guanylyl cyclase (sGC)[30]. Most NO effects are mediated by the activation of sGC, generation of cGMP, and activation of PKG[33]. While sGC functions as an NO sensor, it also promotes angiogenesis in endothelial cells[34]. In the present study, NO may directly inhibit inflammasome activation in BMDMs. Activation of the NLRP3 inflammasome was attenuated in the BMDMs after treatment with an NO donor, but not with an sGC stimulator. Another possibility is a vascular endothelial disorder that increases ROS generation by uncoupling eNOS and NADPH oxidase activity to induce inflammasome activation[6, 35]. These pathways are potential candidates for inflammasome activation in this mouse model.

In conclusion, the absence of the eNOS-NO pathway does not affect inflammasome activation in the physiological condition. However, in terms of pathophysiology, impairment of the eNOS-NO pathway accelerates inflammasome activation. we revealed that endothelial dysfunction, especially the impairment of the eNOS–NO pathway, accelerates the progression of renal tubulointerstitial injury via inflammasome activation. Clinically, patients who develop vascular endothelial dysfunction caused by lifestyle diseases, such as hypertension or diabetes, are known to be at risk for CKD[36]. Our study suggests that the eNOS–NO pathway could be a therapeutic target for the treatment of chronic kidney disease associated with endothelial dysfunction.

## Supporting information

S1 Fig Hydralazine did not suppress inflammasome activation in primary bone marrow-derived macrophages (BMDMs). In an in vitro experiment, inflammasome activation in BMDMs was evaluated by IL-1β secretion into the supernatant. Cont, BMDMs without stimulation; LPS+ATP, BMDMs stimulated with ATP after LPS priming; LPS+ATP+Hyd, BMDMs treated with hydralazine. Data are shown as mean ± SEM. *P < 0.05 vs. Cont.
(TIF)

S2 Fig Systolic and diastolic blood pressure on time points. (A) Systolic blood pressure obtained at 3 time points (0, 2, and 4 weeks after aldosterone infusion). Ald; aldosterone Data are shown as means. *P < 0.05 vs. wild type (WT). (B) Diastolic blood pressure obtained at 3 time points (0, 2, and 4 weeks after aldosterone infusion). Data are shown as means. *P < 0.05 vs. WT.
(TIF)

S3 Fig Histological changes in glomerulus. (A) Periodic acid–Schiff (PAS) staining showing glomerulus morphology. (B) The glomerulosclerotic index was calculated using the glomerular sclerosis score. Data are shown as mean ± SEM. *P < 0.05 vs. WT-vehicle. ^†^P < 0.05 vs. WT-Ald. WT-vehicle, wild type mice administered vehicle only; WT-ald, WT aldosterone infused mice.
(TIF)

S4 Fig Oxidative status in WT and eNOSKO mice administered aldosterone. Nitrotyrosine staining was performed to assess oxidative stress. Bar = 100 μm. WT, Wild type mice; eNOSKO, endothelial nitric oxide synthase knockout mice; WT-Ald, WT aldosterone infused mice; eNOSKO-Ald, eNOSKO aldosterone infused mice.
(TIF)

S1 Table Primer and probe sequences used for quantitative polymerase chain reaction. (DOCX)
