# Supplementary material for: The eNOS-NO pathway attenuates kidney dysfunction via suppression of inflammasome activation in aldosterone-induced renal injury model mice
Source: PLoS One. 2018 Oct 3;13(10):e0203823. doi: 10.1371/journal.pone.0203823 (PMC6169882; doi:10.1371/journal.pone.0203823)
Supplement: S1 Table — (DOCX) [file pone.0203823.s005.docx]

| **S1 Table. Primer and probe sequences used for quantitative polymerase chain reaction.** | | |
| --- | --- | --- |
| Gene | Accession number | Primer and TaqMan probe sequences (5'‑3') |
| F4/80 | NM_010130 | Forward primer: CCTGGCTTTGCATCTAGCA |
|  |  | Reverse primer: AGGAGCCTGGTACATTGGTG |
|  |  | TaqMan probe: FAM‑ TTGATGAGTGCACCCAAGATCCA ‑TAMRA |
| Caspase1 | NM_009807 | Forward primer: ACCCTCAAGTTTTGCCCTTT |
|  |  | Reverse primer: CCCTCGGAGAAAGATGTTGA |
|  |  | TaqMan probe: FAM‑ CCACTCGTACACGTCTTGCCCTCA ‑TAMRA |
| IL-1β | NM_008361 | Forward primer: AGGGCTGCTTCCAAACCT |
|  |  | Reverse primer: TGCCACAGCTTCTCCACA |
|  |  | TaqMan probe: FAM‑ CCTGGGCTGTCCTGATGAGAGCA ‑TAMRA |
| IL-18 | NM_008360 | Forward primer: AGACAGCCTGTGTTCGAGGA |
|  |  | Reverse primer: AGAGGGTCACAGCCAGTCC |
|  |  | TaqMan probe: FAM‑ CAAAGTGCCAGTGAACCCCAGACCA ‑TAMRA |
| NLRP3 | NM_145827 | Forward primer: CCTTGGACCAGGTTCAGTGT |
|  |  | Reverse primer: AGGCAGCAGTTCACCAGTCT |
|  |  | TaqMan probe: FAM‑ TCCAGACACTCATGTTGCCTGTTC ‑TAMRA |
| αSMA | NM_007392 | Forward primer: CAGGCATTGCTGACAGGAT |
|  |  | Reverse primer: GTTCTGGAGGGGCAATGAT |
|  |  | TaqMan probe: FAM‑ CTCGCACCCAGCACCATGAAGA ‑TAMRA |
| CTGF | NM_010217 | Forward primer: TACCGTGGGAGGAACTATCC |
|  |  | Reverse primer: CTCACCTCAGTGTGCGTTCT |
|  |  | TaqMan probe: FAM‑ CAGTTGTTCATTAGCGCACAGTGCC ‑TAMRA |
| FN1 | NM_010233 | Forward primer: ATGATGAGGTGCACGTGTGT |
|  |  | Reverse primer: TGACGCTTGTGGAACGTGT |
|  |  | TaqMan probe: FAM‑TCGTGGAGAATGGGCATGCA‑TAMRA |
| 18S rRNA | NR_003278 | Forward primer: CCTGCGGCTTAATTTGACTC |
|  |  | Reverse primer: GACAAATCGCTCCACCAACT |
|  |  | TaqMan probe: FAM‑ TCTTTCTCGATTCCGTGGGTGGTG ‑TAMRA |
| FAM, 6‑carboxyfluorescein; TAMRA, N,N,N',N'‑tetramethyl‑6‑carboxyrhodamine derivative. | | |
| Gene, genes used for qPCR; Accession number, locus of mRNA; Primer and TaqMan probe sequences, sequences of primer and probe. | | |
